# *Auritidibacter ignavus*, an Emerging Pathogen Associated with Chronic Ear Infections

**DOI:** 10.3201/eid3001.230385

**Published:** 2024-01

**Authors:** Sophie Roth, Maximilian Linxweiler, Jacqueline Rehner, Georges-Pierre Schmartz, Sören L. Becker, Jan Philipp Kühn

**Affiliations:** Saarland University Institute of Medical Microbiology and Hygiene, Homburg/Saar, Germany

**Keywords:** Auriditibacter sp., Auritidibacter ignavus, bacteria, otitis externa, antimicrobial drug resistance, rare pathogen, persistent infection, ear infection, otitis, bacterial infection, Germany

## Abstract

We describe detection of the previously rarely reported gram-positive bacterium *Auritidibacter ignavus* in 3 cases of chronic ear infections in Germany. In all 3 cases, the patients had refractory otorrhea. Although their additional symptoms varied, all patients had an ear canal stenosis and *A. ignavus* detected in microbiologic swab specimens. A correct identification of *A. ignavus* in the clinical microbiology laboratory is hampered by the inability to identify it by using matrix-assisted laser desorption/ionization time-of-flight mass spectrometry. Also, the bacterium might easily be overlooked because of its morphologic similarity to bacterial species of the resident skin flora. We conclude that a high index of suspicion is warranted to identify *A. ignavus* and that it should be particularly considered in patients with chronic external otitis who do not respond clinically to quinolone ear drop therapy.

*Auritidibacter ignavus* is an aerobic gram-positive, rod-shaped bacterium that was described by Yassin et al. in 2011 after isolation from an ear swab specimen (*1*). Thus far, all published cases with microbiological detection of *A. ignavus* were associated with ear infection that clinically manifested as otitis externa with otorrhea, which indicates a specific role of this pathogen in inflammatory diseases of the outer ear (*1*–*3*). However, only a limited number of cases have been published, and scant data are hampering valid conclusions on the clinical relevance and therapeutic implications of this pathogen. In addition, there are discrepant results with regard to susceptibility testing (*1*,*2*).

We describe 3 cases of patients with otorrhea caused by *A. ignavus* detected during March 2021 and October 2022 at the Saarland University Institute of Medical Microbiology and Hygiene (Homburg/Saar, Germany); the total number of ear swab specimens analyzed for diagnostic purposes in the institute’s microbiology laboratory during 2021 and 2022 was 922. We provide an in-depth description of the clinical isolates, including their antimicrobial drug susceptibility patterns and strain comparison by whole-genome sequencing. Furthermore, we review the available literature pertaining to *A. ignavus*.

## Case Reports

**Figure 1:**
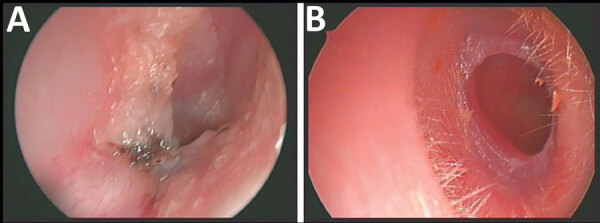
Right ears of 2 patients with chronic ear infections who were infected with *Auritidibacter ignavus*, Germany*.* A) Patient 1. Auditory canal was swollen and red and contained fungal spores. B) Patient 2. Fibrotic stenosis in the cartilaginous part of the ear canal, which was suggestive of a postinflammatory acquired atresia of the external auditory canal.

Written informed consent was obtained from the 3 patients to publish this case report. Patient 1 was a 50-year-old man who sought care for a chronic right-sided otorrhea caused by treatment-resistant external otitis, which had caused symptoms for several months. An outpatient topical treatment with ciprofloxacin ear drops for several weeks did not result in clinical improvement. At initial examination, the patient described persistent itching and otalgia on the affected ear. Clinical examination showed an extensive stenosis of the external auditory canal caused by multiple exostoses that narrowed the lumen by >50%. The ear canal appeared swollen and red by ear microscopy (Figure 1, panel A). The eardrum was covered with black fungal spores. Microbiological wound swab specimens showed *A. ignavus* and the dematiaceous fungus *Exophiala dermatitidis*. Thus, an alternating topical therapy with povidone-iodine drops and ethanol drops was initiated. Four weeks later, the patient reported major clinical improvement and absence of any symptoms. The examination showed a dry ear canal without any abnormal findings.

Patient 2 was a 72-year-old woman who sought care for slowly progressing conductive hearing loss of the right ear and occasional otorrhea. She denied any pain, dizziness, or tinnitus. Although an otologic examination of the left ear showed unremarkable findings, the right side showed a fibrotic, moist auditory canal with stenosis, which was suggestive of a postinflammatory acquired atresia of the external auditory canal (Figure 1, panel B). Audiometry showed an air bone gap of up to 20 dB on the right side with bilateral sensorineural normacusis. To exclude middle and inner ear affection or malformations, computed tomography was performed and showed a partial obstruction of the right external auditory canal by fibrous tissue without any additional pathologic findings. To widen the external auditory canal and to help with outer ear drainage, we performed a meatoplasty. Because the otorrhea did not subside postoperatively, we obtained a microbiological swab specimen, which grew *A. ignavus.* A topical therapy with ethanol drops and nourishing oil drops led to a long-lasting improvement of symptoms without recurring otorrhea.

Patient 3 was a 76-year-old man who had lichen planus and sought care for recurrent otorrhea of both ears for >2 months. He reported no otalgia, vertigo, or tinnitus. A symmetric presbycusis had remained unchanged for years and was treated with conventional hearing aids. On examination, both auditory canals were moist and constricted, clinically manifesting as inflammatory meatal fibrosis, a common finding in patients who have lichen planus. Result of a computed tomography scan showed a bilateral circumferential bony overgrowth of the osseous external auditory canal. A microbiological swab specimen led to the identification of *A. ignavus* in both ears. Thus, a topical therapy with ethanol drops and a tincture of isopropyl alcohol, glycerin, acetic acid, and peppermint oil was initiated. At follow-up after 3 weeks, both auditory canals were dry and without signs of acute infection but with an unchanged fibrotic stenosis.

## Microbiological Characteristics of *A. ignavus*

**Figure 2:**
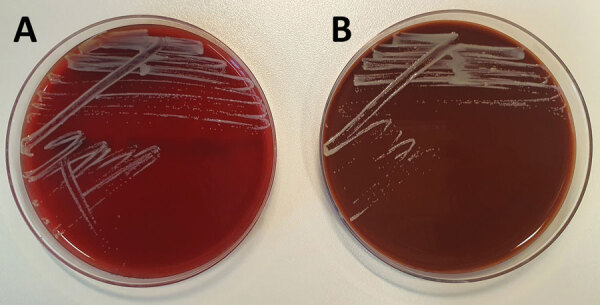
Small white-gray colonies of *Auritidibacter ignavus* in a sample from a chronic ear infection patient, Germany. Colonies are shown after 2 days of incubation at 37°C on tryptic soy blood agar (A) and chocolate agar (B).

In all 3 cases, microbiological ear swab specimens were subjected to standard microbiological culture methods (i.e., incubation on tryptic soy blood agar and chocolate agar for >48 hours). After 1 day of incubation, small white-gray colonies appeared (Figure 2), which changed to a gray-yellow appearance with a slimy surface over the course of few days. On Gram staining, gram-positive rods were observed, with a partially coccoid morphology.

No distinct identification was achieved by matrix-assisted laser desorption/ionization time-of-flight mass spectrometry (Bruker Daltonics). Thus, we performed a 16S broad-range PCR and subsequent Sanger sequencing. Analysis using a BLAST search (https://www.ncbi.nlm.nih.gov/BLAST) based on the National Center for Biotechnology Information genome database showed a sequence homology >99% for *A. ignavus* in all 3 cases.

We performed antimicrobial susceptibility testing using epsilometry on Mueller-Hinton agar with 5% sheep blood. In the absence of specific species-related clinical breakpoints for *A. ignavus*, we assessed the MICs by using the non–species-related breakpoints put forth by the European Committee on Antimicrobial Susceptibility Testing (https://www.eucast.org). We consistently noted high MICs for ciprofloxacin, which are likely to be associated with clinical failure of this drug. In contrast, all isolates were susceptible to β-lactam antimicrobial drugs and vancomycin (Table).

**Table:** Antimicrobial drug susceptibility patterns for 10 drugs of 3 *Auriditibacter ignavus* isolates from patients with chronic ear infections, Germany*

Isolate	MIC, mg/L									
	PEN	CRX	AMS	MEM	VAN	LIN	CLI	DOX	SXT	CIP
1	0.38	0.5	0.25	1.5	0.064	0.5	32	0.5	0.094	32
2	0.19	0.094	0.125	0.38	0.125	0.75	2	0.064	0.008	16
3	0.125	0.125	0.25	0.5	0.064	0.38	1.5	0.125	0.19	12
*Testing was performed by using epsilometry on Mueller-Hinton-Agar with 5% sheep blood. AMS, ampicillin/sulbactam; CRX, cefuroxime; CIP, ciprofloxacin; CLI, clindamycin; DOX, doxycycline; LIN, linezolid; MEM, meropenem; PEN, penicillin, SXT, trimethoprim/sulfamethoxazole; VAN, vancomycin.										

**Figure 3:**
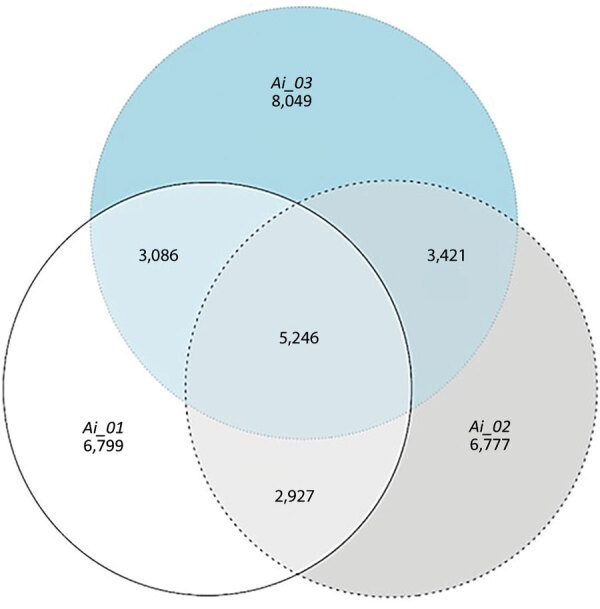
Venn diagram showing overlapping single-nucleotide polymorphism information among *Auritidibacter ignavus* isolates (Ai_01, Ai_02, and Ai_03) from 3 chronic ear infection patients, Germany.

We extracted whole-genome DNA from isolates of *A. ignavus* by using the ZymoBIOMICS DNA Miniprep Kit (Zymo Research Corp.). We performed subsequent whole-genome sequencing by using Illumina PE150 (HiSeq), conducted by Novogene UK Ltd.. We performed quality control of sequencing output by using Fastp version 0.23.2 and MultiQC version 1.13a. We aligned reads against the reference genome of *A. ignavus* (CP031746.1 *Auritidibacter* sp. NML130574) by using Bowtie2 version 2.4. Variant calling using Freebayes version 1.3.2, filtering using Vcftools version 0.1.16 with a set threshold of 20, and comparison with Vcftools suggested that all 3 isolates were unrelated and had only 5,246 single-nucleotide polymorphisms in common (Figure 3).

## Discussion

*Auritidibacter* spp. infections have rarely been reported in the literature. A systematic PubMed/MEDLINE search using the search term “*Auritidibacter*” yielded only 3 results. In 2011, Yassin et al. (*1*) provided a detailed account of this bacterium with a microbiological, biochemical, and phylogenic characterization. The phenotypic culture morphology pattern described in their work matched our own observations. Eight years later, Seth-Smith et al. (*3*) published a complete genome assembly of an isolate from Switzerland and compared it with 4 global genomes, which showed a high diversity within the species. That finding is consistent with our findings of only 24.4%–29.1% single-nucleotide polymorphism identity between the 3 different isolates from the 3 case-patients (Ai_01, 29.1%; Ai_02, 24.4%; Ai_03, 26.5%). More recently, Bernard et al*.* (*2*) investigated 4 isolates of the genus *Auriditibacter* by microbiological and biochemical detection methods, as well as whole-genome sequencing, to assess their relatedness to the species *A. ignavus*.

All of those studies reported only little clinical data of the included patients. We present a report that includes details on the patients’ clinical courses, including the clinical treatment response. Whereas no clear associations of *A. ignavus* infections with predisposing factors was found, outer ear canal stenosis was observed in all 3 patients. This anatomic feature seems to favor the colonization and probably also the infection with this pathogen. However, limited data make it difficult to explicitly establish a causal link between both conditions. Thus, additional studies or case series of a larger number of patients, including a control group of patients with ear canal stenosis and no clinical symptoms suggestive of acute inflammation, would be necessary to distinguish between colonization and infection.

According to Yassin et al*.* (*1*), *A. ignavus* is usually susceptible to β-lactam antimicrobial drugs, whereas Bernard et al*.* (*2*) reported resistance to cefepime. Such discrepancies might partially be explained by different antimicrobial testing methods, which underscores the need for coordinating testing recommendations for rare bacteria such as *A. ignavus*. Particular attention should be paid to our observation of ciprofloxacin resistance in all isolates, a finding that is consistent with the report by Bernard et al*.* (*2*).

Ciprofloxacin ear drops are commonly prescribed in clinical practice. Although MICs enable only limited conclusions on the clinical effectiveness of local antimicrobial drug therapy, we suggest that patients with therapeutic failure after empiric topical treatment with ciprofloxacin ear drops should be assessed for *A. ignavus* by using microbiological tests. The clinical suspicion should be reported to the microbiology laboratory because there is a serious risk of overlooking *A. ignavus* caused by its morphologic similarity to bacterial species belonging to the residential skin flora.

No specific request for an in-depth analysis was made by the treating clinicians in the cases we describe. Thus, increased awareness among the clinical microbiologists was caused by the repeated receipt of ear swab specimens from the patients with the clinical information otorrhea in context with the bacterial growth of presumed physiologic flora in large quantities, which led to a low threshold to submit bacterial colonies to additional testing for species identification. Finally, the absence of *A. ignavus* in matrix-assisted laser desorption/ionization time-of-flight mass spectrometry databases poses an additional threat to correct identification in the clinical microbiology laboratory, as has been reported for other pathogens (*4*).

In conclusion, *A. ignavus* is a novel, potentially underrecognized pathogen that seems to be associated with a distinct clinical pattern in patients with ear infections. A high level of disease awareness and accurate microbiological diagnostics are required for correct identification. In patients who have a clinical course of chronic external otitis and who do not respond to empirical treatment with quinolone ear drops, *Auritidibacter* infection should be considered and further investigated.
